# Vibration upshot of operating mechanical sewing machine: an insight into common peroneal nerve conduction study

**DOI:** 10.1186/s40557-017-0164-1

**Published:** 2017-03-24

**Authors:** Prakash Kumar Yadav, Ram Lochan Yadav, Deepak Sharma, Dev Kumar Shah, Niraj Khatri Sapkota, Dilip Thakur, Nirmala Limbu, Md Nazrul Islam

**Affiliations:** 1 Department of Physiology, Chitwan Medical College, Bharatpur, Nepal; 2 Department of Physiology, BP Koirala Institute of Health Sciences, Dharan, Nepal

**Keywords:** Mechanical sewing machine, Peroneal nerve, Vibration, Latency, Amplitude, Neuropathy

## Abstract

**Background:**

Most of the people associated with tailoring occupation in Nepal are still using mechanical sewing machine as an alternative of new technology for tailoring. Common peroneal nerves of both right and left legs are exposed to strenuous and chronic stress exerted by vibration and paddling of mechanical sewing machine.

**Methods:**

The study included 30 healthy male tailors and 30 healthy male individuals. Anthropometric variables as well as cardio respiratory variables were determined for each subject. Standard Nerve Conduction Techniques using constant measured distances were applied to evaluate common peroneal nerve (motor) in both legs of each individual. Data were analyzed and compared between study and control groups using Man Whitney *U* test setting the significance level *p* ≤ 0.05.

**Results:**

Anthropometric and cardio respiratory variables were not significantly altered between the study and control groups. The Compound muscle action potential (CMAP) latency of common peroneal nerves of both right [(11.29 ± 1.25 vs. 10.03 ± 1.37), *P* < 0.001] and left [(11.28 ± 1.38 vs. 10.05 ± 1.37), *P* < 0.01] legs was found to be significantly prolonged in study group as compared to control group. The Amp-CMAP of common peroneal nerves of both right [(4.57 ± 1.21 vs. 6.22 ± 1.72), *P* < 0.001] and left [(4.31 ± 1.55 vs. 6.25 ± 1.70), *P* < 0.001] legs was found significantly reduced in study group as compared to control group. Similarly, the motor nerve conduction velocity (MNCV) of common peroneal nerves of both right [(43.72 ± 3.25 vs. 47.49 ± 4.17), *P* < 0.001] and left [(42.51 ± 3.82 vs. 46.76 ± 4.51), *P* < 0.001] legs was also found to be significantly reduced in study group in comparison to control group.

**Conclusion:**

Operating mechanical sewing machine by paddling chronically and arduously could have attributed to abnormal nerve conduction study parameters due to vibration effect of the machine on right and left common peroneal nerves. The results of present study follow the trend towards presymptomatic or asymptomatic neuropathy similar to subclinical neuropathy.

## Background

Tailoring is one of the major occupations adopted by skilled people with lower socioeconomic status and education level in Nepal. Nepal is one of the under developed countries in South Asia and the use of new technologies and innovations are out of access for people living in this country [1, 2]. Most of the people associated with tailoring occupation are still using mechanical sewing machine as an alternative of new technology for tailoring [3].

Somatic nerve conduction studies (NCS), which specifically measure the conduction velocity, latency and amplitude of the neurological response following electrical stimulation of peripheral nerve, was applied to assess neural functions. Nerves of lower limbs including common peroneal nerve are exposed to chronic stress imposed by chronic paddling and vibration generated by operation of mechanical sewing machine [4].

Nerve conduction studies are an essential part of the work-up of peripheral neuropathies. Many neuropathic syndromes can be suspected on clinical grounds, but optimal use of nerve conduction study techniques allows diagnostic classification and is therefore, crucial to understanding and separation of neuropathies [5].

The common peroneal nerve is superficial as it courses around the fibular neck. Because of its location, it is highly susceptible to injury. Compression of the peroneal nerve at the fibular head usually manifests as “foot drop.” Weakness of dorsiflexion and eversion may result in patient complaints of falling because they have caught their toes while climbing stairs or walking on thick rugs [6].

Nerve conduction study (NCS) helps in delineating the extent and distribution of neural lesions. It enables clinicians to differentiate the two major groups of peripheral diseases: demyelination and axonal degeneration [7].

It should be considered that some factor such as the functional overload generated by physical exercise, positively contribute to higher MNCV [8, 9]. We should remember that physical exercises, besides causing alterations in the musculoskeletal structure, also cause alterations in the functioning of the motor units, increasing for example, its excitability [10].

Health effects associated with Whole Body Vibration (WBV) have been well documented and include low-back pain, spinal degeneration, neck problems, headaches, nausea, gastrointestinal tract problems, disturbed sleep, and autonomic nervous system dysfunction [11, 12]. HAV exposure causes a condition referred to as hand-arm-vibration syndrome (HAVS). HAVS is associated with vascular, neurological, and musculoskeletal problems of the hand-arm system [13].

In vibration-associated neuropathy, conceivable target structures could be peripheral sensory receptors, large or thin myelinated nerve fibers, and the small-caliber, non-myelinated C fibers. Electrophysiological studies aimed at defining the nature of the vibration injury have provided conflicting results [14].

Exposure to Foot Transmitted Vibration (FTV) may cause an analogous syndrome in the lower extremities; however, little is known about the characteristics of occupational FTV or clinical implications with prolonged exposure. The extent of disability caused by vibration exposure is variable and may depend on the magnitude, frequency, and duration of exposure [15].

Tailors operating mechanical sewing machine by paddling for long-term is a source of high intensity vibration exerted on both legs and that effect might cause structural or functional changes in nerves of both right and left legs, and most probably common peroneal nerve. Therefore, this study was designed to check the effect of repetitive and chronic vibration imposed on nerve conduction study parameters of common peroneal nerves (velocity, latency and amplitude) of Nepalese tailors.

## Methods

### Subjects

The study was conducted in Neurophysiology Laboratory of Department of Basic and Clinical Physiology, BP Koirala Institute of Health Sciences (BPKIHS), Dharan (during 2011–2012). The study population included 30 healthy tailors (study group) and 30 healthy volunteers (control group). Average duration of occupation in the study group was more than 12 years. Subjects ranged in age from 18 to 60 years with average of 35 years. None of the subjects were suffering from any known neuromuscular or musculoskeletal diseases, cardiovascular diseases, and respiratory diseases and were not taking any drugs which affect nerve conduction study. Subjects from both study and control groups were not indulging in smoking or drinking. All the participants provided written informed consent that had been previously approved by the institutional ethical committee. The ethical approval for the study was obtained from the institutional ethical committee. Subjects who passed the initial screening based on clinical history or physical examination were selected for motor nerve conduction studies (MNCSs).

### Methods

Prior to the study, all subjects were informed of the study procedure, purposes, and known risks and thereby obtained their informed consent. This study was conducted according to the guidelines of the Declaration of Helsinki and approved by the institutional ethical committee, BPKIHS, Dharan, Nepal. The sites of stimulation and recording of common peroneal nerve are shown in the (Table 1). The nerve tested was common peroneal nerve (both right and left). Digital Nihon Kohden Machine (NM-420S, H636, Japan) with its accessories was used for nerve conduction studies. The temperature of the recording room was kept around 26 ± 2 °C_._ Anthropometric and cardio-respiratory parameters of the subjects were recorded as shown in the (Tables 2 and 3).

**Table 1:** Site of stimulation and recording

Motor Nerve	Site of stimulation			Recording site
	Proximal 2	Proximal 1	Distal	
Common peroneal	Lateral popliteal fossa	Below fibular head: lateral calf	Anterior ankle	Extensor digitorumbrevis

**Table 2:** Comparison of anthropometric and cardio-respiratory variables between study group and control group

Variables	Study group(*n* = 30)	Control group(*n* = 30)	*P* value
	Mean ± SD	Mean ± SD	
Age (yrs)	35.07 ± 4.95	34.2 ± 3.94	0.651
Wt (kg)	63.83 ± 4.14	65.07 ± 3.79	0.300
Ht (m)	1.63 ± 0.04	1.64 ± 0.04	0.200
LORL (cm)	84.13 ± 3.38	83.7 ± 5.08	0.370
LOLL (cm)	84.13 ± 3.38	83.7 ± 5.08	0.370
BMI (kg/m^2^)	23.92 ± 1.79	23.94 ± 1.43	1.00
SBP(mmHg)	120.67 ± 2.95	118.73 ± 4.28	0.099
DBP(mmHg)	78.67 ± 3.72	79 ± 3.47	0.513
PR(beats/min)	72.83 ± 3.40	71.6 ± 2.62	0.15
RR(cycles/min)	16.2 ± 1.86	16.47 ± 1.22	0.567

*P* ≤ 0.05 = statistically significant

*Wt* Weight, *Ht* Height, *BMI* Body mass index, *LORL* Length of right leg, *LOLL* Length of left leg, *SBP* Systolic blood pressure, *DBP* Diastolic blood pressure, *PR* Pulse rate, *RR* Respiration rate

**Table 3:** Comparison of Right and Left common peroneal nerves conduction study variables between study group and control group

Variables		Study group (*n* = 30)	Control group (*n* = 30)	*P* value
		Mean ± SD	Mean ± SD	
Right	PL-CMAP(ms)	11.29 ± 1.25	10.03 ± 1.37	<0.001
	DL-CMAP(ms)	3.70 ± 0.58	3.07 ± 0.58	<0.001
	Amp(P)-CMAP(mv)	4.57 ± 1.21	6.22 ± 1.72	<0.001
	AMP(D)-CMAP(mv)	5.45 ± 1.69	6.91 ± 2.05	0.006
	Dist.PDSS(cm)	329.67 ± 19.91	328.33 ± 20.01	0.86
	MNCV(m/s)	43.72 ± 3.25	47.49 ± 4.17	<0.001
Left	PL-CMAP(ms)	11.28 ± 1.38	10.05 ± 1.37	0.001
	DL-CMAP(ms)	3.26 ± 0.40	2.75 ± 0.37	<0.001
	Amp(P)-CMAP(mv	4.31 ± 1.55	6.25 ± 1.70	<0.001
	AMP(D)-CMAP(mv)	4.83 ± 1.71	6.76 ± 2.10	<0.001
	Dist.PDSS(cm)	336.67 ± 24.54	334.0 ± 25.94	0.598
	MNCV(m/s)	42.51 ± 3.82	46.76 ± 4.51	<0.001

*P* ≤ 0.05 = statistically significant

*PL-CMAP* Proximal latency compound muscle action potential, *DL-CMAP* Distal latency, *Amp(P)-CMAP* Amplitude proximal, *AMP(D)-CMAP* Amplitude distal, *Dist.PDSS* Distance between proximal and distal stimulating sites, *MNCV* Motor nerve conduction velocity

### Common peroneal nerve conduction study

**Fig. 1:**
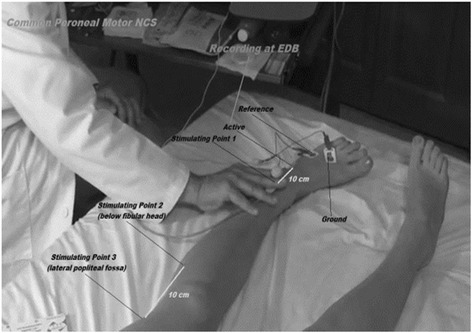
Localization of stimulating and recording electrodes used during common peroneal nerve conduction study. Source: Basic principles of nerve conduction study and electromyography. In: Misulis KE, Head. TC editors. Essentials of clinical neurophysiology. Burlington; p. 127-160, 2003

For motor nerve conduction study, the stimulator using adhesive tape was used for surface stimulation. It was placed on the skin overlying the nerve at two or more sites along the course of the nerve after cleaning the site with skin purifier. Before applying a brief pulse of current, ground electrode was placed between the stimulating and recording electrodes. The recording and reference electrode were placed using belly tendon montage with the recording electrode placed over the mid belly of the respective muscle, as close to the estimated end plate site as possible and the reference to the tendon at a minimum distance of 3 cm. The sites of stimulation and recording electrodes for common peroneal nerve are shown in (Table 1) and (Fig. 1) [16].

**Fig. 2:**
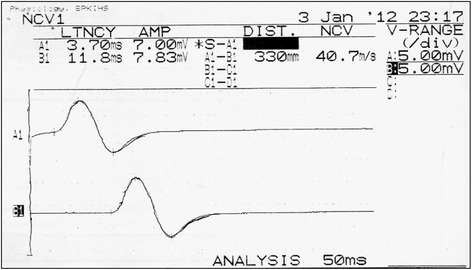
Representative trace of motor (common peroneal) nerve action potential of right and left legs

Stimulation of the nerve being studied was accomplished using a brief burst of direct electric current. The gain was set at 5 mv per division. Stimulation duration was in the range of 0.2 ms and the amount of current never exceeded more than 50 mA because it was the upper limit available in the machine. The current of the stimulator was initially set to zero, then gradually increased with successive stimuli. A compound muscle action potential (CMAP) appeared that grew larger with the increasing stimulus strength (Fig. 2). Current was increased to the point that CMAP no longer increased in size, from that point the current was increased by another 20% to ensure supra-maximal stimulation.

### Statistical analysis

The SPSS package (Statistical Package for Social Sciences, Version-20, and Chicago, Illinois, USA) for personal computer was used for the statistical analysis. Shapiro-Wilk’s W test was applied to examine normality in the distribution of data. Results are presented as mean ± standard deviation (SD). The differences in variables between the groups were tested using Student’s *t*-test. A *p* ≤0.05 was considered significant.

## Results

Both the tailors (study group) and healthy volunteers (control group) formed a very homogenous population without significant differences in age, height, weight, length of right and left legs and body mass index (Table 2). Resting heart rate, systolic blood pressure, diastolic blood pressure and respiration rate were not significantly different in study group compared to control group as shown in (Table 2).

Common peroneal nerve (motor) conduction study showed significantly lower proximal and distal compound muscle action potential (CMAP) amplitudes and prolonged CMAP proximal and distal latencies in both right and left legs of study group as compared to control group as shown in (Table 3). Further, motor NCS of the same nerve revealed significantly lower motor nerve conduction velocity in both right and left legs of study group as compared to control group as shown in (Table 3).

Right and Left common peroneal motor nerve:

## Discussion

In the present study, we evaluated the motor nerve conduction study parameters of both right and left common peroneal nerves of tailors (study group) and normal healthy volunteers (control group). CMAP latencies of common peroneal nerves of both right and left legs were found significantly prolonged (*P*˂0.001) in tailors, whereas its amplitudes and conduction velocities were found to be significantly low (*P*˂0.001) in them as compared to those of controls. Our results did not show any significant difference in anthropometric variables-age, weight, height, BMI, length of right and left legs. Similarly, cardio-respiratory variables- SBP, DBP, PR and RR were also comparable between the study and control groups. Therefore, these factors could not have caused any net effect on the nerve conduction study parameters of common peroneal nerves of both right and left legs.

### CMAP-Latency

Right and left common peroneal CMAP proximal and distal latencies were significantly high in study group in comparison to control group. Our results are quite in agreement with the result of Feinberg et al.[17]. According to their studies, there was increase in CMAP latency due to peripheral nerve injuries in some of hockey players, but in contrast with the study conducted by Budaket al. [18]. They found that there was decrease in CMAP latency in their studies of lower extremities in long distance runners. Also, our findings are in contrast with the studies conducted by several researchers [19, 20]. According to their studies there was positive effect of physical training on nerve conduction parameters. In another study, there was also decreased nerve conduction in the workers occupationally exposed to mechanical vibration [21]. Murata K et al. showed that there was effect of vibration in peripheral nervous system and found negative impact of vibration on nerve conduction in workers of vibrating tool industry [22]. Latency is a reflection of the activation pattern of individual neurons comprising the peripheral nerve. In the compound action potential produced in a nerve conduction test, the fastest arriving fibers (the most myelinated) determine the onset latency and hence the velocity of that nerve. Prolonged latencies reflect the dropout of faster myelinated fibers and result in slower nerve velocities [23, 24].

### CMAP-Amplitude

Our results showed that right and left common peroneal CMAP-Amplitude (proximal and distal) were found significantly low in the study group in comparison to control group. Our results are similar to the results of the study conducted by Chatterjee et al. [25]. They found that there was reduced CMAP-Amplitude in rock-drillers and reduced muscle mass as well. Although our findings are in contrast with the study ofRoss et al. [26]. They found positive effect of hypertrophied muscles on CMAP-Amplitude of peroneal nerves in long distance runners. Reduced nerve conduction amplitude in the presence of a normal nerve conduction velocity would indicate a dropout of smaller-diameter axons in the nerve, accounting for the decreased amplitude of the compound muscle action potential (CMAP) while maintaining a normal nerve conduction velocity. The lack of change of the nerve conduction velocity would indicate that the axonal injury has not affected the faster (most myelinated) fibers contained in the nerve bundle [27].

Selective injury of fiber types can be observed in various hereditary neuropathies. By contrast, nerve compression injuries cause damage at the point of compression and can involve a mixed population of large- and small-diameter axons resulting in a decrease in both the amplitude and the latency of the evoked nerve response (representing an axonal, demyelinating polyneuropathy) [28].

The earlier portion of the evoked peak represents the larger-diameter fibers and the latter portion of the response, progressively smaller-diameter fibers. Collectively, the height of the nerve conduction amplitude is a representation of all the axon bundles comprising the nerve [29]. Injury to axons within the nerve bundle will result in a dropout in the total number of axons actually stimulated in the nerve bundle and hence a reduction in the amplitude of the evoked compound motor action potential [30]. Therefore, reduction of the nerve conduction amplitude indicates the degree of axonal injury in the nerve bundle [5, 31].

### Motor nerve conduction velocity (MNCV)

Our results showed that right and left common peroneal MNCV were found significantly reduced in study group when compared to control group. There are reports in the literature that strength and muscle power athletes present higher motor nervous conduction velocity (MNCV) than endurance athletes, despite not having significant difference between these modalities, as well as that the MNCV of trained individuals is greater than in untrained and injured individuals. It has also been reported that the MNCV is greater in the dominant limb (D_L_) when compared with the non-dominant limb (N_DL_) in trained subjects [32, 33] On the other hand, it has been mentioned that hypertrophy of muscles adjacent to the nervous tract of the dominant limb of trained individuals may lead to compression of the nerve and consequent reduction of MNCV [34].

According to Untunen et al. there was polyneuropathy and muscle weakness in operators with vibration syndrome who had been working on vibrating tools for a long time [35].

The pathophysiology of vibration-induced neuropathy is complex, but includes morphological changes, such as degeneration of nerve fibres and fibrosis [36, 37]. Stromberg et al. reported pathological changes in biopsies from the posterior interosseus nerve in patients exposed to vibration, and concluded that demyelination might be the primary lesion in neuropathy following vibration exposure [38].

Studies performed on rats simulating vibration from hand-held power tools, reported axonal damage and myelin fragmentation in nerves that had been exposed to vibration, explaining the observed increased stress response in the vibration exposed rat nerves [39–42].

Most studies show a reduction in conduction velocity, indicating demyelination, of the fastestlarge myelinated fibres and in later stages, when the patients have more pronounced symptoms, a lower amplitude, indicating a loss of nerve fibers [43].

Vibration of mechanical sewing machine could have overshadowed the effect of regular exercise of lower limbs by chronic paddling in tailors. Poor nerve conduction study (NCS) parameters (L-CMAP, Amp-CMAP and MNCV) of peroneal nerves of both right and left legs in study group as compared to control group could be due to vibration effect of mechanical sewing machine. Most probably the effect of exercise (regular paddling) has not been able to counter the detrimental effect of vibration on lower limbs [22].

## Conclusion

In conclusion our study showed that the reduction in amplitude and conduction velocity of CMAP of common peroneal nerves in study group whereas increase in latency of CMAP of corresponding nerves in the same group as compared to control group might be due to vibration effect of mechanical sewing machine. Vibration effect of mechanical sewing machine possibly overweighed the exercising effect mechanical sewing machine on lower limbs in tailors thereby suggesting trends towards subclinical neuropathy of common peroneal nerves. Other studies on large sample size are suggested to be conducted to confirm the findings of our study and reveal the cause of subclinical neuropathy as well. Furthermore, a new study is required to delineate the magnitude of the problem and to better characterize and control foot-transmitted vibration as an occupational hazard.

## Abbreviations

AMP(D)-CMAP: Amplitude distal
Amp(P)-CMAP: Amplitude proximal
BMI: Body mass index
BP: Diastolic blood pressure
CMAP: Compound muscle action potential
Dist.PDSS: Distance between proximal and distal stimulating sites
DL-CMAP: Distal latency
Ht: Height
LOLL: Length of left leg
LORL: Length of right leg
MNCV: Motor nerve conduction velocity
PL-CMAP: Proximal latency compound muscle action potential
PR: Pulse rate
RR: Respiration rate
SBP: Systolic blood pressure
Wt: Weight
